# Undernutrition among under-five indigenous Mbororo children in the Foumban and Galim health districts of Cameroon: a cross-sectional study

**DOI:** 10.11604/pamj.2021.38.352.25030

**Published:** 2021-04-12

**Authors:** Florence Titu Manjong, Vincent Siysi Verla, Thomas Obinchemti Egbe, Dickson Shey Nsagha

**Affiliations:** 1 Department of Public Health and Hygiene, Faculty of Health Sciences, University of Buea, Buea, Cameroon,; 2 Department of Pharmacy Technology, St Louis University Institute of Health and Biomedical Sciences, Bamenda, Cameroon,; 3 Department of Obstetrics and Gynecology, Faculty of Health Sciences, University of Buea, Buea, Cameroon,; 4 Department of Internal Medicine and Pediatrics, Faculty of Health Sciences, University of Buea, Buea, Cameroon

**Keywords:** Stunting, wasting, underweight, under-five children, indigenous, Mbororo

## Abstract

**Introduction:**

despite increasing research interest on Indigenous peoples´ health worldwide, the nutritional status of Indigenous children in Cameroon remains unknown. This study was conducted to assess the prevalence of stunting, wasting, and underweight among under-five Indigenous Mbororo children in the Foumban and Galim health districts of the West Region.

**Methods:**

a cross-sectional study was conducted involving 472 child-caregiver pairs from 16 Mbororo Communities in the Foumban and Galim health districts. Interviewer-administered questionnaires were used for data collection. Anthropometric measurements were collected using standard procedures. Socio-demographic data were analyzed using descriptive statistics. Anthropometric indices: height-for-age, weight-for-height, and weight-for-age - z-scores were analyzed using z-score 06 Stata version 11 and compared with World Health Organization growth reference standards. Ethical approval was obtained from the Faculty of Health Sciences Institutional Review Board of the University of Buea.

**Results:**

overall prevalence of stunting, wasting and underweight were 55.08% (95% CI: 50.5-59.58), 13.77% (95% CI: 10.65-16.89), and 31.99% (95% CI: 27.76-36.21), respectively. Severe stunting, wasting and underweight were 34.53% (95% CI: 30.22-38.83), 3.18% (95% CI: 1.58-4.76), and 10.59% (95% CI: 7.80-13.37), respectively. Rates of stunting, wasting and underweight for female and male were: 56.88% and 52.71%; 12.38% and 14.72%; and 30.73% and 32.55%, respectively. Stunting, wasting and underweight rates varied with child age.

**Conclusion:**

the prevalence of undernutrition was high, indicating a serious public health problem and the necessity for strategies to ensure the optimal health of the target population.

## Introduction

Adequate nutrition is vital in early childhood to ensure optimal growth, development, and survival [1,2]. Nevertheless, poor diets and resulting malnutrition are among the greatest health and societal challenges of our time [3]. Children under five years of age are most vulnerable to malnutrition, particularly undernutrition [4,5]. Global estimates reveal that stunting and wasting affected 144 million (21.2%) and 47 million (6.9%) under-five children respectively in 2019 [6]. Despite substantial progress in undernutrition reduction globally, stunting and wasting rates remain inadmissibly high in developing countries [6]. Africa and Asia shoulder the largest share of stunting (Africa 40%, Asia 54%), and wasting (Africa 27%, Asia 69%) [6]. Africa is the only region where the number of stunted children increased from 49.7 million in 2018 to 57.5 million in 2019 [6]. In Cameroon, child undernutrition rates have been trending upwards over the past two and half decades [7]. The prevalence of stunting, wasting, and underweight increased from 24.4% to 32%, 3% to 5.2%, and 13.6 % to 14.8%, respectively from 1990 to 2014 [7]. There are equally significant within-country inequalities in the magnitude of undernutrition rates [8]. The magnitudes of these estimates show that undernutrition is an important public health problem.

Undernutrition undermines the very survival of children, accounting for 45% of under-five mortality globally [9]. While wasting increases the risk of infant mortality, stunting is associated with long-term cognitive impairment, poor school performance, and low economic productivity [1,10-12]. Child undernutrition is consequently a threat to sustainable development, justifying the Sustainable Development Goals (SDG) 2.2 of reducing all forms of malnutrition by 2030 [13,14]. Achieving this target particularly in the present context of COVID-19 [6] will largely hinge on continuous efforts and comprehensive preventive policies targeting evidence-based high-risk groups [15].

Among high-risk groups are Indigenous peoples numbering more than 370 million in some 90 countries in the world [16]. They disproportionately experience poorer health status than their non-Indigenous counterparts [17-20]. In Cameroon, the Indigenous Mbororos are an ethnic minority and marginalized population [21]. They constitute a significant proportion of the population of the West region. As nomadic pastoralists, they live in geographically isolated and hard-to-reach rural settings where malnutrition rates are disproportionately higher in Cameroon [8]. Despite their vulnerability to malnutrition, the nutritional status of Mbororo children in the region remains unknown. Moreover, the persistent problem of child undernutrition in Cameroon may not be tackled adequately if high-risk sub-population disparities are not thoroughly assessed. It is against this backdrop that this study was conducted to fill the knowledge gap. Our baseline data will provide evidence for growth monitoring as well as inform strategies to ensure optimal health of the target population.

## Methods

**Study area and design**: a community-based cross-sectional study was conducted from August to September 2019, as part of a larger study entitled “Assessing malnutrition and associated determinants among under-five Indigenous Mbororo children in two health districts of the West Region of Cameroon”. The region has an estimated population of 1,785,285 distributed over a surface area of 13,960 km^2^ [22]. Agriculture and commercial businesses are major sources of livelihood for the population. The region is host to people of diverse ethnicities, including the Indigenous Mbororo peoples who reside in larger communities in 7 (Bangouraim, Bangante, Foumban, Foumbot, Kouoptamo, Galim, and Mbouda) of the 20 health districts of the region. Foumban health district (FHD) and Galim Health District (GHD) were randomly selected for the study.

**Study population and inclusion criteria**: the study comprised of Mbororo children and their female primary caregivers as respondents. Included in the study were 0-59 months old male and female children; caregivers with 6 months minimum residence status and caregivers who gave verbal or written informed consent and parental assents to participate in the study. Those who were seriously sick and those not meeting the inclusion criteria were excluded from the study The Mbororos are an ethnic minority group and Indigenous peoples in Cameroon [21], with very distinctive customs and cultural identities. They are predominantly Muslims and speak *“Fulfulde”* language. As pastoralists, their livelihood is animal production, compelling them to live in isolated hard-to-reach rural settings where cattle grazing land is readily available [21]. Nevertheless, with the influence of modernization and other cultures, strong traditional customs and practices are changing [21]. There is an on-going shift from nomadic to a more sedentary lifestyle, and from pastoralists to agro-pastoralists [20].

**Sample size determination**: the minimum sample size (n) of 384 was calculated using single population proportion formula:

$$n_{0}=\frac{z^{2}pq}{e^{2}}$$

Where Z is 1.96 at 95% confidence level, P is anticipated prevalence of 32% (national mean for stunting) [8], q is 1-p, e (5% or 0.05) is the margin of error and considering a 10% non-response rate.

**Figure 1:**
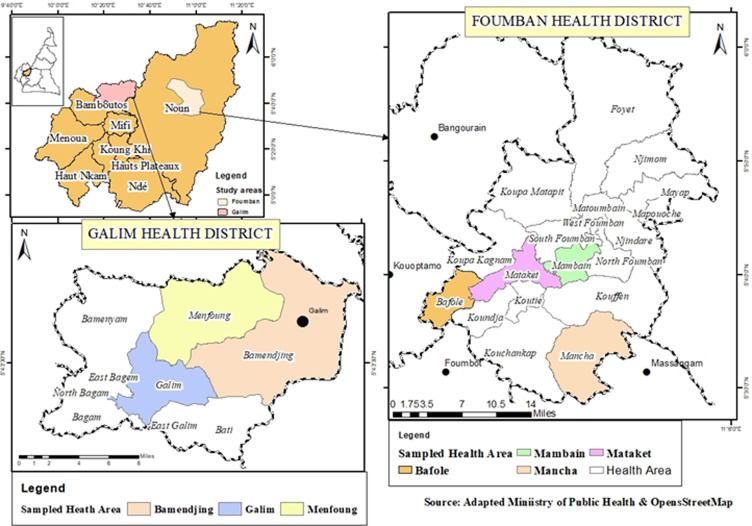
map of study area

**Sampling procedure**: a multi-stage sampling technique was used. FHD and GHD were randomly selected by balloting from 7 health districts with the highest population of the Mbororos. In the second stage, Bafole, Mataket, Mambain, Mancha, Galim, Menfung, and Bamenjing with sizable Mbororo communities were purposively selected. A sample of 16 Mbororo communities was selected from 22 listed communities by balloting. Geographically accessible households with children 0-59 months were identified and listed for each community. Using probability proportionate to size, 472 child-caregiver pairs were selected from 636 eligible households and enrolled for the study. Exhaustive sampling was employed for smaller communities and systematic random sampling for larger communities. For the households with 2 or more eligible children, one child was selected randomly by ballot (Figure 1).

**Study variables**: the variables were stunting, wasting, and underweight, defined as height-for-age, weight-for-height, and weight-for-age, respectively, z-score < -2 standard deviations (SD) from the reference WHO Child Growth Standards mean [23]. Covariates were child´s age (in months) categorized as (0-5), (6-11), (12-17), (18-23), (24-35), (36-47), (48-59), and child sex categorized as male and female.

### Data collection

**Training of data collectors and field supervisors**: ten undergraduates and graduates (including 6 Mbororo females) were recruited based on their proficiency in French, English, and *Fulfulde*, familiarity with the Mbororo culture, and prior experience with surveys. They underwent a two-day intensive training employing adult learning approaches that included didactic sessions, demonstrations, question and answer sessions, and role-playing. The content of the training manual adapted from a guide to anthropometry) [24] included procedures for obtaining participants´ consent/assent; tools and procedures for interviews and anthropometric measurements.

**Piloting of data collection instruments**: structured interviewer-administered questionnaires adapted from the UNICEF MICs/ tool [25] were prepared in English and translated and back-translated into French and *“Fulfulde”*. They were pre-tested for language, clarity of content among non-participating child-mother pairs (38) with similar characteristics to the study population in the neighboring Mbouda health district. Necessary corrections and modifications were done. Portable anthropometry tools (battery-powered digital infant and toddler weighing scales, stadiometer, measuring tapes, and lying wooden boards) were equally pre-tested for reliability.

**Sociodemographic data collection**: household, caregiver, and child sociodemographic data were collected through face-to-face interviews with caregivers. Each interview took 45 to 60 minutes and was conducted at the interviewee´s convenient time, day, and venue and language preference. The interviewers read out the questions to the respondents and completed the questionnaires accordingly.

**Anthropometric measurements**: the weight and height/length measurements were performed using standard procedures [23-26]. All children wore light clothing, were barefooted and not wearing hats, hair, and body ornaments. Lying/sitting weights for 0-23 month´s children were measured to the nearest 0.01 kg, and standing weights for older children were measured to the nearest 0.1 kg. Weighing scale was calibrated to zero before each measurement. Recumbent lengths for 0-23 month´s children were measured to the nearest 0.1 cm with measuring tapes and lying boards placed on a flat ground surface. Standing heights for older children were measured to the nearest 0.1 cm, with head, shoulder, buttock, and heel touching the vertical surface of the stadiometer. Measurement was taken in duplicates and the mean recorded.

**Data management and statistical analysis**: raw data were checked, edited, coded, and fed into Microsoft Excel version 13 spreadsheet and exported to Stata version 11 for analysis. Socio-demographic data were analyzed using descriptive statistics. Three anthropometric indices: height-for-age (HAZ), weight-for-height (WHZ), and weight-for-age (WAZ), Z-scores were computed and compared with the World Health Organization 2006 growth WHO standard median [23]. Results were presented as tables and charts. All analysis was performed at 95% confidence interval and p < 0.05 level of significance.

**Ethical considerations**: an ethical approval (ref: 2019/1002-07/UB/SG/IRB/FHS) was obtained from the Faculty of Health Sciences-Institutional Review Board (IRB) of the University of Buea. Supporting administrative authorization was obtained from the West Regional Delegation of Health. Informed verbal and signed consent/assents were obtained from participants before inclusion in the study. Participation was voluntary, and participants were informed on their right to withdraw from the study at any time. Anonymity and confidentiality were assured and maintained.

## Results

**Socio-demographic characteristics of caregivers**: of the 472 caregivers enrolled, the mean age was 28.11±7.57 years and 93.22% were 18 years and above. Their mean height and weight were 149.2±10.47 cm and 51.26±30.23 kg, respectively. The majority (99.35%) resided in the rural settings, 92.09% were married, and the mean age at first birth was 17.2 ±2.98 years. Almost half (49.36%) had not attended formal education, 74.15% were unemployed and 78.76% were financially dependent (Table 1).

**Table 1:** socio-demographic characteristics of caregivers

Variables/ categories	Pool	FHD	GHD	Chi Square Test	P-value
**Age**					
<18 years	32(6.78)	13(4.05)	19(12.58)	11.831	0.001
≥18	440(93.22)	308(95.95)	132(87.42)		
**Marital Status**					
Single	13(2.78)	9(2.82)	4(2.68)		
Monogamy married	256(54.70)	171(53.61)	85(57.05)		
Polygamy married	175(37.39)	123(38.56)	52(34.90)		
Widow	14(2.99)	8(2.51)	6(4.03)		
Divorced/separation	10(2.14)	8(2.51)	2(1.34)	2.019	0.732
**Educational status**					
No formal education	231(49.36)	160(50.16)	71(47.65)		
Primary education	182(38.89)	113(35.42)	69(46.31)		
Secondary education	55(11.75)	46(14.42)	9(6.04)	9.292	0.010
**Occupation**					
House wife	347(74.15)	230(72.10)	117(78.52)		
Farmer	29(6.20)	14(4.39)	15(10.07)		
Commercial business	62(13.25)	50(15.67)	12(8.05)		
Healthcare provider	1(0.21)	1(0.31)	5(3.36)		
Teaching	2(0.43)	2(0.63)	--		
Administrator	1(0.21)	1(0.31)	--	14.074	0.029
Others	26(5.56)	21(6.58)	--		
**Major Sources of Income**					
Agriculture income	37(8.19)	18(5.79)	19(13.48)		
Salary earns	1(0.22)	1(0.32)	-		0.005
Animal breeding	4(0.88)	2(0.64)	2(1.42)	14.878	
Commercial income	54(11.95)	46(14.79)	8(5.67)		
Others	356(78.76)	244(78.46)	112(79.43)		
**Residence**					
Semi-rural	3(0.65)	2(0.65)	1(0.67)	0.001	0.974
Rural	456(99.35)	308(99.35)	148(99.33)		
Mean age	28.11(Min: 12; Max: 60; SD:7:57)				
Mean age at first delivery	17.178 (Min:11, Max:29, SD: 2.98				
Mother´ height(in cm)	149.2 , Min : 45.1, Max: 170.1, SD: 10.47				
Mother´ Weight(in kg)	51.26, Min : 30.2, Max:104, SD: 30.23				

**Sociodemographic characteristics of under-five Mbororo children**: of the 472 children enrolled, 53.81% were males and 46.19% were females. Age range was 0-59 months, 6.62% was less than 6 months and 93.28% were 6 months and above, with the majority (36.11% and 26, 5%) within 24-47 and 48-59 months age brackets. The mean age was 29.87 ± 17.98 months, mean of 79.12± 17.75cm and mean weight was 12.42 12.86 ± 12.86kg (Table 2).

**Table 2:** socio-demographic characteristics of under-five Mbororo children

Variables/ categories	Pool	FHD	GHD	Chi Square Test	P-value
Sex					
Male	254(53.81)	152(50.17)	75(52.82)	0.272	0.602
Female	218(46.19)	151(49.83)	67(47.18)		
**Age Group (months)**					
0 - 5	31(6.62)	20(6.31)	11(7.28)	2.692	0.611
6 - 11	65(13.89)	41(12.93)	24(15.89)		
12 - 23	79(16.88)	59(18.61)	20(13.25)		
24 - 47	169(36.11)	115(36.28)	54(35.76)		
48 - 59	124(26.50)	82(25.87)	42(27.81)		
Mean age(months)	29.87 SD:17.98; Min: 1 months; Max: 60 months				
Mean weight (kg)	12.42 SD: 12.86; Min: 3 kg; Max: 28 kg				
Mean height/length ( cm)	79.12 SD: 17.95; Min: 6.2 cm; Max: 113.6				

**Prevalence of stunting, wasting and underweight in under-five Mbororo children**: the prevalence of stunting, wasting, and underweight were 55.08% (95% CI: 50.58-59.58), 13.77% (95% CI: 10.65-16.89), and 31.99% (95% CI: 27.76-36.21), respectively. The prevalence of severe stunting, wasting, and underweight recorded were 34.53% (95% CI: 30.22-38.83), 3.18% (95%CI: 1.58-4.76), and 10.59% (95% CI: 7.80-13.37) (Table 3).

**Table 3:** prevalence of stunting, wasting and underweight among under-five Mbororo children

Anthropometric Indices	Categories	Frequency (n)	Percent (%)	95% C.I	
				**Lower Bound**	**Upper Bound**
**Stunting**	Stunting (−2SD)	260	55.08	0.5058	0.5958
	Stunting (−3SD)	163	34.53	0.3022	0. 3883
**Wasting**	Wasting (−2SD)	65	13.77	0.1065	0.1689
	Wasting (−3SD)	15	3.18	0.0158	0.0476
**Underweight**	Underweight (−2SD)	151	31.99	0.2776	0.3621
	Underweight (-3SD)	50	10.59	0.0780	0.1337

**Figure 2:**
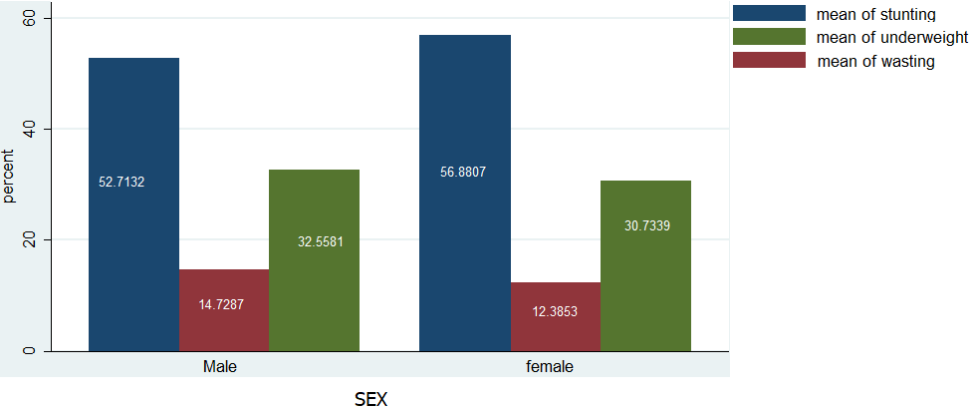
prevalence of stunting, wasting and underweight in under-5 Mbororo children by child´s sex

**Prevalence of stunting, wasting and underweight in under-five Mbororo children by child´s sex**: the prevalence of stunting was higher in females (56.88) than in males (52.71). The rate of wasting in males (14.72%) was higher compared to females (12.38%). Likewise, the prevalence of underweight was higher in males (32.55%) than the female (30.73%) (Figure 2).

**Figure 3:**
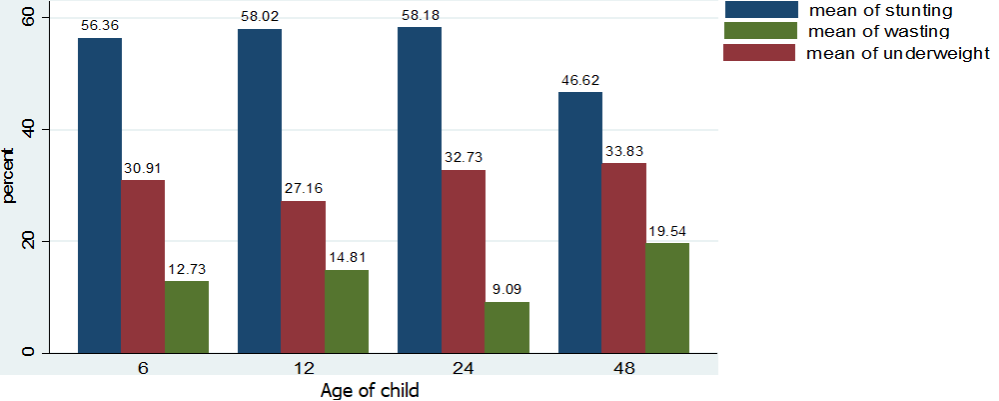
prevalence of stunting, wasting and underweight among study participants by child´s age

**Prevalence of stunting, wasting and underweight in under-five Mbororo children by child´s age**: stunting prevalence steadily increased from 56.36% for 0-5 months and peaked at 58.18% at 48 months. Underweight rates were high among children 0-5 months (30.91%), 12-23 months (32.73%), and highest at 24 months (33.8%). The highest prevalence of wasting was observed among children 0-5months (12.73%), 6-11 months (14.81%), and children above 48 months (19.54%) (Figure 3).

## Discussion

Stunting (55.08%), wasting (13.77%) and underweight (31.99%) rates in this study were much higher than national estimates for stunting (32%), wasting (5%) and underweight (15%), and the West regional estimates for stunting (30.6%), wasting (0.8%) and underweight (4.8%) [8]. According to WHO standards, these rates are considered as “very high” public health significance [11], triggering intervention. Comparable and higher stunting rates of 55.7%, 61%, and 83.8% were reported among under-five Indigenous children in India [27-29]. Whereas, several studies have shown lower stunting rates such as 25.7% and 28.2% among Indigenous children in Malaysia [30], 45.8% in Batouri, Cameroon [31], 43.1% and 39.9% and 39.9% in Ethiopia [32,33]. The high stunting rate observed in this study could partially be attributed to the low socioeconomic status of the caregivers. The caregiver illiteracy rate was nearly 50%, 74.15% were unemployed and 78.76% were financially dependent. Based on gender, stunting was higher in female children (56.88%) compared to their male counterparts (52.71%), in contrast with national estimates [8] and previous studies among Indigenous children India, Malaysia, Guatemala [27,29,30,34] and non-Indigenous children in Cameroon, Ghana, Ethiopia and Zambia [35-38]. A conceivable reason for higher rates of stunting in male children could be that girls are generally less active and stay closer to their mothers at home, while boys engage in high physical activities that expend high amounts of energy that would have been channeled into increasing growth [39-41]. However, this may not apply to the Mbororo population characterized by male dominance and gender inequalities that demands more physical household work from the girl child compared to the boy child.

Stunting rates were equally shown to increase with child´s age, consistent with evidence from other Indigenous populations in Brazil, India, and Malaysia [28-30] and non-Indigenous populations in Ghana and Zambia [36,38]. This pattern is not unanticipated considering that increasing child age is characterized by intense growth and physical activity, with correspondingly high energy demands which if not adequately met will result in linear growth deficits. The proportion of wasting (13.33%), far exceeds rates of 3.2% and 11.3% in Cameroon [31,35], 5.4%, 5.3% in Ghana [36] and 5.4% and 1.3% among Indigenous children in Brazil [28,42] and 11.8% in a nomadic population in Ethiopia [43]. Contrary to our findings, other authors in Malaysia [30] and Ethiopia [32] observed higher wasting proportions of 52% and 16.2% respectively. The high rate of wasting in our study could be related to childhood infectious diseases such as diarrhea and malaria, typical of resource-limited settings in Cameroon. Malaria for instance is the most significant cause of child morbidity and mortality in Cameroon [8]. Our study further revealed a higher proportion of wasting in male children (14.72%) than female children (12.38%). This finding is in agreement with the national estimates and [8] and a study in Ethiopia [37]. It is generally assumed that male children eat more than female children, and will tend to more malnourished if not adequately fed. The prevalence of wasting was equally higher among children less than 12 months and peaked at 48 months and above. According to national statistics, wasting is lowest among children 0-5 months and highest among children 6-17 months [8]. Likewise, Boah and colleagues in Ghana [36] showed a higher wasting proportion in 6-11 months children. Dapi and others in Banja, Cameroon concluded that wasting is higher in children below 30 months than older children [35]. Wasting in younger children could be attributed to the repeated incidence of diarrhea episodes resulting from enteric pathogens in contaminated food, water, and environment, heightened by child exploratory behavior such as crawling, sucking and mouthing of objects [44,45]. Moreover, younger children are more susceptible to childhood infections owing to a weaker immune system compared to older children.

Our estimate for underweight (31.99%) is similar to a 30.2% rate reported by Nagahori and colleagues in Batouri, Cameroon [31], but is five times higher than the national mean (5%) [8]. However, some authors have observed much lower rates: 5.2% and 6.67% in Cameroon [35,46], 15.8% and 24.8% in Ethiopia [32,33]. Still, not in agreement with our findings are reported higher rates of 32.7%, 37%, 64%, 50%, and 38.15% among Indigenous populations in India, Malaysia and Nepal [28-30,47,48], 47.7% in pastoral communities in Ethiopia [37] and 39.5% by Khan and colleagues in Pakistan (39.5%,) [49]. As a composite of stunting and wasting, a high proportion of underweight in this study could be due to the possible reasons advanced for stunting and wasting above. Based on gender underweight rates were higher in males (32.55%) than the females (30.73%), consistent with national statistics. Contrary, Ngondi, and colleagues [50] in Cameroon showed higher underweight rates in female children (31.5%), compared to their males (30.6%) counterparts. The results further revealed that the prevalence of underweight was higher in children above 24 months, in agreement with the national estimates [8] and evidence from Ghana [36] and Nepal [47]. This could be because poor hygienic practices among toddlers lead to frequent infections and repeated episodes of diarrhea, and consequent weight loss [30]. This could apply to the study population, considering their predominantly rural residence (99.35%) characterized by conditions that increase susceptibility to childhood infections.

**Strengths and limitations of the study**: the strength of this study lies in its representative sample and high participation rate. Additionally, anthropometric measurements were performed using standard procedures. However, a few limitations should be considered when interpreting the findings. First, recall bias regarding the mother´s recall of child´s month and year of birth could not be adequately reduced due to a lack of supporting records. Second, the distinct socio-cultural, religious, and linguistic characteristics of the target population were a constraint to effective communication. This was minimized by involving female Mbororo and non-Mbororo data collectors who are fluent in *“Fulfulde”* and familiar with the Mbororo culture.

## Conclusion

The prevalence of undernutrition among the study population was significantly high, signifying a critical public health problem. Stunting and wasting prevalence varied with age and were higher in female and male children respectively. These findings underscore the urgent need for further research to identify the contributing risk factors to adequately inform targeted interventions.

### What is known about this topic

- The national burden of malnutrition in Cameroon;
- Several studies have reported the nutritional status of under-five children in varying settings in Cameroon.

### What this study adds

- To our best knowledge, this is the first study to report the nutritional status of the Indigenous Mbororo children in the West Region of Cameroon;
- Besides providing empirical current baseline data for future reference, our findings form the basis for monitoring and evaluation of ongoing national child malnutrition control programs;
- Our results highlight the urgent need for further studies to identify the predictors of undernutrition to inform intervention efforts.
